# Platelet desialylation and TFH cells–the novel pathway of immune thrombocytopenia

**DOI:** 10.1186/s40164-021-00214-5

**Published:** 2021-03-15

**Authors:** Yuwen Chen, Jianda Hu, Yingyu Chen

**Affiliations:** Department of Hematology, Fujian Provincial Key Laboratory of Hematology, Fujian Institute of Hematology, Fujian Medical University Union Hospital, No.29 Xinquan Road, 350001 Fuzhou, Fujian China

**Keywords:** Immune thrombocytopenia, Platelet, Desialylation, T follicular Helper cells

## Abstract

Immune thrombocytopenia (ITP) is an autoimmune disease characterized by immune-mediated destruction of one’s own platelets. The progression of thrombocytopenia involves an imbalance of platelet production and clearance. B cells can induce autoantibodies, and T cells contribute to the pathological progression as well. Some patients with ITP have a poor response to common first-line therapies. Recent studies have shown that a novel Fc-independent platelet clearance pathway is associated with poor prognosis in these patients. By this pathway, desialylated platelets can be cleared by Ashwell-Morell receptor (AMR) on hepatocytes. Research has demonstrated that patients with refractory ITP usually have a high level of desialylation, indicating the important role of sialylation on platelet membrane glycoprotein (GP) in patients with primary immune thrombocytopenia, and neuraminidase 1(NEU1) translocation might be involved in this process. Patients with ITP who are positive for anti-GPIbα antibodies have a poor prognosis, which indicates that anti-GPIbα antibodies are associated with this Fc-independent platelet clearance pathway. Experiments have proven that these antibodies could lead to the desialylation of GPs on platelets. The T follicular helper (TFH) cell level is related to the expression of the anti-GPIbα antibody, which indicates its role in the progression of desialylation. This review will discuss platelet clearance and production, especially the role of the anti-GPIbα antibody and desialylation in the pathophysiology of ITP and therapy for this disease.

## Background

Immune thrombocytopenia (ITP) is a common clinical bleeding disorder characterized by increased platelet clearance and decreased platelet production. A recent study also described a novel type of thrombocytopenia classified by the appearance of giant platelets and variable neutropenia [1]. This disease may cause acute and chronic bleeding. Interestingly, Gialluisi et al. identified platelet distribution width (PDW) as a new potential biomarker of depression and psychopathology [2], for the first time describing the relationship between thrombocytopenia and emotions. ITP is generally regarded as a primary autoimmune condition [3], yet in some cases, it is observed after other diseases, such as infections [4, 5], chronic lymphocytic leukemia and many other autoimmune conditions [5]. Studies have found that platelet glycoprotein (GP)IIb/IIIa and GPIb/IX are the two most frequently targeted autoantigens and are associated with Fc-FcγR interactions and Fc-independent platelet activation. Hereditary macrothrombocytopenia is mainly caused by mutations in the genes coding for GPIb/IX and non-muscle myosin heavy chain [6, 7], and a defect in glycosylation can also lead to macrothrombocytopenia [8]. T cells, especially T follicular helper cells (TFHs), play an important role in the pathophysiology as well. However, the detailed progression remains poorly understood, and there are no confirmed diagnostic or prognostic biomarkers. Recently, several studies have found a relationship between anti-GPIb/IX-promoted platelet desialylation and the Fc-independent pathway, identifying platelet desialylation as a critical reporter of disease progression or healing. Common first-line therapies for ITP include immunosuppressive and immunomodulatory agents, such as corticosteroids, intravenous immunoglobulin G (IVIG) and anti-RhD treatment [3, 9]. Recombinant human thrombopoietin (TPO) and vindesine are feasible therapies as well [10]. Splenectomy has been considered the last resort for patients who have no response to therapy [11]. However, approximately 15–20% of patients are refractory to first-line therapy, and approximately 10% are refractory to splenectomy [12, 13]. In recent years, some reports have demonstrated that antibody specificity may play an important role in the response to therapy [14]. The discovery of the desialylation-induced novel Fc-independent pathway indicates the potential therapeutic effect of inhibitors of sialidases; however, the exact curative effect requires further research. This review will provide an overview of the pathophysiological mechanisms involved in the progression and treatment of this disease.

## Pathophysiology of ITP

Platelets are essential for hemostasis, and a steady platelet supply is necessary to avoid spontaneous bleeding. In other words, platelet clearance and production are closely related to the occurrence of bleeding disorders.

### Platelet clearance

#### Autoantibodies

Several mechanisms mediate platelet clearance, and B cell-induced autoantibodies targeting platelet GPs were suggested to be the main cause of platelet clearance [15]. Autoantibodies can be detected in approximately 50% of patients with ITP [16]. Among them, approximately 70–80 % of patients have antibodies against GPIIb/IIIa, approximately 20–40% have antibodies against the GPIb complex, and some patients have antibodies against both or other GPs [9].

Current theories suggest that autoantibodies against platelet GPIIb/IIIa-mediated platelet destruction in the spleen via macrophages through Fc-FcγR interactions [9] and are responsive to the reticuloendothelial system [17], primarily in the spleen. These antibodies may also target the precursors to platelets and megakaryocytes (MKs) [18]. Anti-GPIIb/IIIa antibodies may suppress the agglutination and adhesiveness of platelets [19], which results in platelet clearance.

The GPIb complex, however, has a completely different structure and function than GPIIb/IIIa; it is a completely distinct platelet receptor. This complex results in the most heavily glycosylated platelet surface with approximately 60% carbohydrate by weight [20]. Binding of GPIbα to von Willebrand factor (VWF) can induce substantial platelet activity by initiating outside-in signaling. Recently, it was shown that anti-GPIbα is closely related to platelet activation and apoptosis [21, 22]. In addition, infusion of monoclonal antibodies (MAbs) targeting the N-terminal ligand binding domain (LBD) of GPIb may lead to a decrease in platelets [18]. A case report has shown that the GPIbα association with FcγRIIa in lipid rafts may induce platelet destruction [21]. Moreover, it has been found that deglycosylation of the complex results in hepatic clearance of platelets [9], which indicates the important role of glycan modification of GPIbα in inducing platelet clearance. Furthermore, some studies have shown that anti-GPIba-mediated desialylation and platelet activation can lead to FcγR-independent clearance via AMR in hepatocytes [9], and the process involves a positive feedback loop that will enhance the effect [19]. The detailed mechanisms will be discussed below.

#### T cells

As a main component in immunity, T cells play an important role in self-defense, and changing the number and function of these cells is associated with the progression of ITP. Several studies have already shown an increased number of Th1, Th17 and Th22 cells and a decrease in CD4 + CD25 + FoxP3 + regulatory T cells (Tregs) in patients with ITP [23–25]. Some findings have suggested that the autologous stimulation of Th1 cells via IL-2 production leads to an increase in auto-Abs [26]. A Th1 dominant imbalance in Th1/Th2 subsets has also been observed in patients with ITP, with increased IL-2 and IFN-γ and reduced IL-4 [27]. The number of functional Tregs was also observed to be significantly decreased in the circulation, spleen and bone marrow [28]. An elevated number of CD3 + T cells has been reported in the bone marrow (BM) of patients [29]. Additionally, recent data have shown that CD8 + cytotoxic T lymphocytes (CTLs) may be involved in the progression of platelet destruction in patients with ITP [30]. It has been proven that cell-mediated cytotoxic lysis can induce an increase in platelet destruction in ITP [31]. Although antibodies are often central to the pathogenesis of ITP, some patients lack detectable platelet autoantibodies and instead have CTLs that induce platelet clearance via apoptosis and perforin/granzyme-mediated cytotoxicity [32, 33]. Moreover, some data have proven that antibody and T-cell attacks can occur at the level of MKs [34]. Interestingly, it was recently shown that MKs can present antigens on their surface in association with MHC class I molecules and lead to the activation of specific CD8 + T cells [35]. BM CD8 + T cells are platelet-specific and activated, which could impair the differentiation of MKs and lead to a decrease in platelet production [36]. CD8 + T cells were also shown to be associated with platelet desialylation and to induce platelet clearance in the liver [37].

TFHs are T cell subsets that play a critical role in B cell differentiation and proliferation [38]. Studies have demonstrated the bidirectional crosstalk between TFHs and B cells and the requirement of B cells for TFH survival. This crosstalk involved the ICOS-L/ICOS and CD40/CD154 signaling pathways [39], and germinal center B cells are essential in the maintenance of TFHs [40]. Some studies have observed the expansion of TFHs during ITP and have proven that B cell depletion can induce a decrease in TFHs and in CXCL13 and IL-21. Additionally, a dramatic decrease was observed after RTX treatment [41]. In addition, a study by Yao et al. has shown a higher percentage of TFHs in patients with ITP [42]. These researchers found that TFHs can cooperate with B cells to produce Abs, and the expression of TFHs is increased in newly diagnosed patients, indicating that TFHs may contribute to the immunopathogenesis of ITP. Furthermore, Xie et al. showed that the frequencies of circulating CD4 + CXCR5 + TFHs with high ICOS and high PD-1 expression and associated molecules such as IL-21 and Bcl-6 were significantly increased in patients with ITP [43]. Moreover, studies have observed a strongly positive correlation between TFHs and platelet GPIbα antibodies, implying that TFHs may be involved in platelet destruction via platelet antibodies [43], and the relationship indicated the role of TFHs in platelet desialylation.

#### Apoptotic factors

Similar to other nucleated cells, platelet apoptosis is another important cause of platelet clearance. Antiapoptotic Bcl-2 family proteins and proapoptotic Bak and Bax are all involved in this process. Caspase-mediated apoptosis is also a critical mechanism [44]. The redistribution of phosphatidylserine is an essential molecular cue for platelet clearance by phagocytes [45]. In addition, mitochondrial outer membrane permeabilization plays a critical role in platelet apoptosis [18]. Moreover, recent findings have implied that antibody-dependent cytotoxicity can induce platelet apoptosis in the progression of ITP [46].

### Platelet production

TPO is a hematopoietic growth factor that is essential for platelet production and is the primary regulator of thrombopoiesis. TPO production is constitutive, and its level is maintained by the metabolism of platelets and MKs [47]. TPO balances the level of platelets via the surface c-Mpl receptor [48]. A mouse model has demonstrated that TPO signaling in MKs is negligible in platelet production, and the key role of TPO is the generation and stimulation of bipotential MK precursors. Mpl expression in MKs and platelets can control platelets by restricting TPO [49, 50]. A study found that GPIbα deficiency can lead to a low level of TPO by decreasing hepatic TPO mRNA transcription and production [48]. This phenomenon may be caused by disruption of Ashwell-Morell receptor (AMR)-desialylated platelet signaling by the JAK1/2 inhibitor [10]. *In vivo* and *in vitro* experiments have demonstrated that sialylated GPIbα will not rescue impaired TPO production, which suggests that GPIbα is a prerequisite for TPO generation independent of desialylation [48]. It has been assumed that antibodies targeting GPIba may block TPO generation and therefore decrease platelet production [19]. In addition, research has proven that IL-6 stimulates TPO mRNA expression, and both IL-6 and desialylated platelets result in STAT3-mediated hepatic TPO mRNA expression downstream of the AMR-JAK2 and IL6 receptor (IL-6R)-JAK1 signaling cascades, respectively [10]. Moreover, thrombocytopenia is a common side effect in myeloproliferative neoplasm patients with JAK1/2 inhibitor therapy, which target in hematopoietic stem and precursor cell mutant JAK2-V617F as well as wile-type JAK2. These findings indicate that JAK2-associated pathway may be involved in the pathophysiology of thrombocytopenia [10].

## Desialylation of platelets

Young platelet surfaces have high levels of sialic acid. Platelet glycocalicins in the membranes, like GPIIb/IIIa and GPIb/IX, are full of carbohydrate. Among them, GPIbα contains the most sugar chains and the terminal of the sugar chains contain a lager quantity of sialic acid. As they circulate, these sialic acid residue in the surface of GPs are gradually removed by sialidases such as neuraminidase 1 and neuraminidase 3, which result in platelet desialylation [51]. As mentioned above, desialylation of platelets is closely related to thrombocytopenia. Recently, several studies have focused on the relationship between platelet desialylation and thrombocytopenia (Table 1). A multicenter observational study has shown an increased platelet desialylation level in septic patients with thrombocytopenia, and the decrease in platelet counts was modified by the desialylation inhibitor oseltamivir [52]. The level of sialic acid residues in platelets was significantly reduced with old age, diabetes, and lymphoma, which indicates the essential role of platelet desialylation in these conditions [53]. A previous study reported that circulating platelets that have been remodeled by pathogen neuraminidase activity can be decreased by the AMR as a host protective mechanism in pneumococcal sepsis [54]. Moreover, Zhang et al. found that prolonged isolated thrombocytopenia patients have lower levels of sialic acids and increased β-galactose exposure, and the researchers concluded that neuraminidase 1(NEU1) translocation, platelet apoptosis, and phagocytosis by macrophages may be associated with this process [55]. It has also been proven that desialylation on cold-storage platelets can induce platelet desialylation and apoptosis via the GPIbα and 14-3-3 proteins [56]. The degree of desialylation is related to the storage duration [57]. Desialylation is detected after arterial events as well and is related to low antithrombin activity [58]. In addition, Stivala et al. reported that platelets treated with amotosalen and ultraviolet A light had significantly increased desialylation, which indicated the relationship between platelet desialylation and platelet function [59]. Moreover, mice deficient in α2,3-sialyltransferase IV showed strong thrombocytopenia [60], indicating that glycosylation defect is restricted to α2,3-sialylation, which leads to a decrease in the expression of sialyl-Lewis X and ligands for selections, myelin-associated glycoprotein and Maackia amurensis lectin II [1].

**Table 1:** Effect of platelet desialylation in thrombocytopenia

Reference	Finding	Methodology
Grodzielski [46]	Desialylation↑in the presence of GPIb-positive ITP	CD34 + cells were incubated with ITP or control plasma
Dupont [63]	Type 2B VWD and mice carrying the p.V1316M mutation elevated desialylation	Samples from patients and mice with type 2B VWD were collected
Jansen [70]	Refrigeration↑→desialylation↑, DANA inhibited desialylation, Desialylation↑→GPIb, GPV shedding↑	Platelets stored in DANA or PBS, fresh or refrigerated at 4 °C, were injected into mice
Kullaya [74]	Desialylation↑→ADP signaling ↑→NanA exposure↑→platelet hyperactivity	Whole blood or PRP was incubated with 200 mU purified neuraminidase and supernatants with and without NanA
Qiu [37]	CD8 + T cells ↑→phagocytosis↑ DANA inhibits phagocytosis	CD8 + T cells and platelets were cocultured, and DANA was added
Grozovsky [73]	Platelets desialylation↑→AMR→JAK2/STAT3↑→TPO mRNA↑	WT or Asgrs/st3gal4- mice were treated with sialydase and RAMPs
Hinek [83]	NEU1→PDGF/IGF receptors→PDGF and IGF↓→arterial SMCs↓	AoSMCs were treated with ddNANA, anti-neuraminidase and preimmune IgG
Ma [79]	SLC35A1 mutations→sialylation↓→megakaryocyte↓, megakaryocyte maturation↓, platelets clearance in liver↑	Mouse line with the Slc35a1 gene was established

Therefore, how does platelet desialylation participate in the progression of ITP? Patients with ITP exhibited significantly increased platelet desialylation [61], and platelets from patients with NR-ITP had lower levels of sialic acid in their surface glycans [62]. Dupont et al. observed abnormally high levels of desialylation in patients with thrombocytopenia and identified GPIb as a desialylation target [63]. Previous studies have demonstrated that when incubated with ITP plasma, control platelet desialylation levels are increased, especially in plasma with anti-GPIb autoantibodies [46]. A case report identified severe platelet desialylation in a patient with GPIb/IX antibody-mediated ITP, which indicates the role of GPIb/IX antibody in platelet desialylation [64]. Novel research has demonstrated in murine models of ITP that anti-GPIb/IX antibodies lead to platelet activation and neuraminidase translocation to the cell membrane, which can induce platelet desialylation and Fc-independent hepatic platelet clearance [65].

**Fig. 1:**
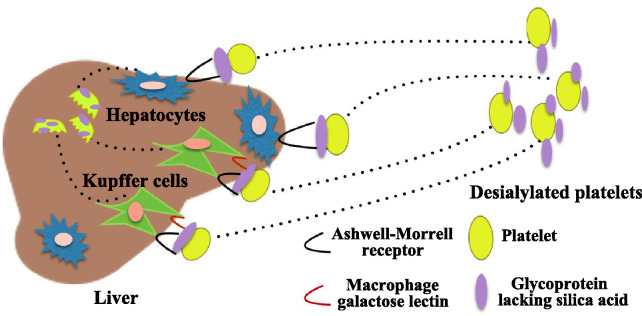
The clearance of desialylated platelets mediated by AMR and MGL. Varied mechanisms contribute to the platelet clearance. The desialylated glycoproteins (GPs) on the platelet surface can be recognized and captured by the Ashwell-Morrell receptor (AMR) of hepatocyte. Desialylated platelets from the circulation may also directly adhere to Kupffer cells. Macrophage galactose lectin (MGL) of Kupffer cells facilitates the phagocytosis and clearance of desialylated platelets through the collaboration of AMR

It has been assumed that patients with ITP with anti-GPIb autoantibodies have a higher level of desialylation, and desialylated platelets can be recognized and cleared by hepatic AMR (Fig. 1). The Ashwell receptor is the major lectin of hepatocytes for asialoglycoproteins [66] and is regarded as an endocytic receptor responsible for the removal of circulating plasma glycoproteins or glycolipids lacking silica acid [67]. This receptor is composed of type 2 transmembrane glycoproteins termed asialoglycoprotein receptor-1 and asialoglycoprotein receptor-2. The ST3Gal family is an enzyme that adds silica acid in α2,3 linkage to galactose residues at the termini of N- and O-glycan chains to mask endogenous asialoglycoprotein receptor (ASGPR) ligands. Studies have concluded that ST3Gal-IV deficiency can cause the exposure of glycans on platelets, leading to recognition by the Ashwell receptor and promotion of platelet clearance by hepatocytes [66]. Li et al.’s study demonstrated that mice lack of O-glycan exhibits a high risk of thrombocytopenia, and hepatic AMR promotes phagocytosis of platelets by Kupffer cells through its C-type lectin receptor CLEC4F [68]. Besides, the other study has proved that Kupffer cells could directly recognize and participate in the phagocytosis of desialylated platelets via intergrin αMβ2 as well [55]. A recent study identified that macrophage galactose lectin (MGL) of Kupffer cells facilitate the clearance of desialylated platelets through the collaboration of AMR [69] (Fig. 1).

Additionally, some studies have shown that desialylation of platelets binding to VWF receptor triggers platelet clearance in patients with ITP [70]. It has already been proposed that platelet action and clearance may be mediated by active VWF [71]. Recently, a report demonstrated that the binding of VWF to platelets in dengue patient can lead to neuraminidase-mediated platelet desialylation and platelet clearance [72, 73]. Recent study results have shown that pneumococcal neuraminidase A can remove sialic acid (desialylation) from the platelet surface, leading to platelet hyperactivity and clearance, and the progression may be associated with the ADP pathway [74]. A study has demonstrated that NEU1 and NEU2 in platelets are highly dependent on VWF-GPIbα, and specific clustering of GPIbα by VWF can trigger platelet clearance via NEU1 and NEU2 [75].

Furthermore, a recent article reported the first case of a patient who had acquired Glanzmann thrombasthenia (GT) due to ITP with FcγRIIa-mediated platelet desialylation independent of platelet activation, which strongly indicated the relationship between platelet desialylation and FcγRIIa [76]. As mentioned above, CD8 + T cells may also induce platelet desialylation [37]. Studies have shown that patients with positive cytotoxicity of CD8 + T cells have a higher desialylation level, suggesting that CD8 + T cells may induce platelet clearance via desialylation [37]. In addition, the relationship between TFHs and GPIbα antibodies deserves more attention, and the role of TFHs in platelet desialylation requires more research. Moreover, the loss of sialic acid residues on the platelet surface is associated with a reduction in the lymphocyte Treg subset, and it has been reported that sialylated antigens can inhibit the generation and function of T cells by regulating dendritic cells [77], which suggests a potential correlation between platelet desialylation and the immune response [78]. It has also been shown that Slc35a1 mutations are closely related to the progression of ITP via platelet desialylation [79].

The defect in α2,3-sialylation may also have an effect on platelet generation [1]. In some ways, desialylation may induce thrombocytopoiesis. It has been demonstrated that aging and refrigeration of platelets can induce the expression of hepatic TPO mRNA and protein via desialylation, and recent studies have found that desialylation can increase the production of TPO in patients with ITP [80]. Desialylation of platelets binding with AMR can stimulate the production of TPO [81]. The AMR-desialylated platelet interaction can lead to activation of the JAK2/STAT3 signaling pathway, stimulating hepatic synthesis and secretion of TPO. This mechanism could induce hepatic TPO expression, providing a feedback mechanism. As more platelets are removed, more TPO is produced [73, 82]. Some studies have shown that desialylation may inhibit platelet production. Some studies have provided direct evidence that neuraminidase will induce the desialylation of platelet-derived growth factor (PDGF) receptors via the mitogenic ligand PDGF-BB and thus diminish intracellular signals [83]. Moreover, studies have proven that Slc35a1 mutations significantly reduced sialylation in megakaryocytes, thus affecting the number and maturation of megakaryocytes from BM [79]. A recent study has also proven that autoantibodies can induce the cleavage of silica acid from not only platelets but also megakaryocytes, and desialylation can lead to impaired platelet adhesion and megakaryocyte differentiation. The results strongly demonstrated that the cleavage of silica acid will affect the production, survival and function of human platelets [84]. However, how sialylation can influence the generation of platelets in BM remains unknown.

Recently, clinical research has identified the relationship between the types of antibodies and the site of platelet clearance via platelet survival studies. The result contradicts the hypothesis that anti-GPIbα destroys platelets in the liver and that therapy inhibits the effect of desialylation in the progression of thrombocytopenia [85]. Additionally, analysis of ITP plasma samples and platelets from healthy controls indicates that there is no correlation between apoptosis and loss of sialic acid [46]. Generally, sialylation strongly affects the number and function of circulating platelets, and desialylation leads to their clearance from the circulation. Desialylation leading to anti-GPIbα-VWF binding triggers platelet clearance through the AMR-JAK2 signaling cascade in the liver, but the detailed mechanism still needs to be established. In addition, CD8 + T cells and TFHs may be associated with the progression of platelet desialylation, which may be a novel mechanism of ITP. In addition, desialylation can impair the function of platelets via FcγRIIa. Moreover, platelet desialylation is linked to platelet activation and apoptosis. Platelet desialylation may also affect the production of platelets, but the details require further elucidation.

## Treatment of ITP

Primary ITP tends to be a chronic disease, and the aim of therapy is to achieve a hemostatic platelet count of approximately 20–30 × 10^9^/L [86] and restore durable platelet function.

### First‐line therapy

The first-line therapies of ITP include corticosteroids, immunoglobulin G (IVIG) and anti-Rh D because they can protect against anti-GPIIb/IIIa autoantibody-mediated thrombocytopenia. Corticosteroids such as dexamethasone and prednisone have been proven to modulate B-cell and dendritic cell activation [3], and up to 80% of patients respond to therapy [87]. Corticosteroids and anti-Rh D, targeting Fc and FcγR-dependent mechanisms, may also be effective methods to restore platelet numbers [9]. IVIG can be used when patients are steroid-resistant and can rapidly increase the platelet count [88]. A study of 19 patients showed a response rate of 75% with IVIG and a rapid increase in platelet count within 1 h in 53% of the patients [89]. Apparently, the effect of first-line treatment is to decrease platelet clearance via Fcγ receptors. These molecules target the neonatal FcR and FcR pathways by acting as a functional blockade of platelet clearance and decreasing the autoantibody half-life [19]. Some patients with ITP responded to first-line therapy, but some relapsed afterward, requiring further treatment.

### Second-line therapy

Up to 70–90% of patients fail to receive the initial therapy and do not achieve complete remission [90]. In these cases, splenectomy would be an efficient treatment because the spleen is the primary site for platelet-related T cell and B cell activation and platelet destruction [91]. Several studies have shown a 65–70% complete response with a 60–70% long-term response [92, 93], and this strategy is a safe option even in patients with very low platelet counts [94]. Rituximab (RTX) is a monoclonal antibody against the CD20 antigen (anti-CD20). This anti-CD20 agent is an effective immunosuppressant [3]. Thus, B cell depletion induced by RTX is also an optional treatment method. A study showed that 50% of patients had an initial response to RTX [3]. In addition, studies showed that combining RTX with dexamethasone resulted in a higher remission rate, with the combination at 63 versus 35 % with monotherapy after 6 months and 53 versus 33% at 1 year. The study also demonstrated the efficacy of RTX alone with an initial response rate of 40–60% and a sustained response rate of 20% at 5 years [95].

TPO-receptor agonists (TPO-RAs), such as eltrombopag and romiplostim, may be an option when first-line treatments fail. Increased platelet counts can be observed in 60–80% of patients with chronic ITP who are refractory to traditional therapy [96, 97]. Such agonists could activate TPO receptors on MKs and induce platelet production by the JAK2 and STAT5 pathways [3]. TPO-RAs may also affect immunomodulation via restoration of the monocyte Fcγ receptor balance and Treg function [98–100]. In addition, some articles demonstrated that CD8 + Tregs may be a potential cellular treatment in autoimmune diseases due to their immunosuppressive function [19] and response to steroid therapy. Moreover, treatments such as dexamethasone and prednisone can contribute to an effective decrease in the frequency of TFHs, indicating that TFHs might be a new therapeutic target for ITP [43]. Recent studies have indicated the efficacy of azathioprine, cyclophosphamide, cyclosporin A, danazol, dapsone, mycophenolate mofetil, vinblastine and vincristine [101], and these studies are still ongoing. Some articles have demonstrated that a high level of IL-8 is associated with an abnormal Th1/Th2 cytokine profile in active ITP, and IL-18 binding protein (IL-18BP) has shown the ability to restore the Th1/Th2 balance, which suggests the therapeutic potential of IL-18BP [102]. In addition, lncRNA GAS5 can relieve ITP by inhibiting Th17 differentiation [103]. Despite the limited efficacy and the risks associated with their use, platelet transfusions remain a possible treatment for patients [104].

### Desialylation and ITP treatment

A multicenter cohort study indicated that the presence of anti-GPIb/IX antibodies is a predictive factor for poor response to first-line treatment [17]. Thus, anti-GPIb-mediated ITP is often refractory to therapies targeting the FcγR pathway or splenectomy. Data have already shown that antisera against GPIbα can induce platelet activation [9], indicating the important role of GPIbα in ITP therapy. Since anti-GPIb/IX antibodies can trigger platelet desialylation, platelet desialylation may be a potential biomarker in assessing refractoriness [19]. Some research data claimed that platelets refrigerated in the presence of inhibitors of sialidases showed improved recovery and circulation [70]. Moreover, some experiments have proven that desialylation-induced Fc-independent platelet clearance can be effectively rescued by 2-deoxy-2,3-didehydro-N-acetylneuraminic acid (DANA). DANA is a synthetic inhibitor of sialylation that can significantly inhibit platelet desialylation induced by anti-GPIb/IX antibodies [105]. These results identified sialylation inhibitors as a potential therapy for patients with ITP [18, 19]. Sialylation inhibitors are believed to delay platelet clearance via the inhibition of desialylation. In addition, a case of acquired GT due to ITP showed that treatment with neuraminidase inhibitors prevents platelet clearance via anti-α β antibodies [76]. A recent report demonstrated that oseltamivir phosphate, a kind of sialidase inhibitor agent, could reduce the desialylation level in patients with anti-GPIb/IX ITP, thus substantially alleviating thrombocytopenia and suggesting sialylation inhibitors as a novel approach for the treatment of ITP [106]. In addition, a previous study suggested that the increase in platelet count is independent of influenza, and the curative effect is associated with the duration of treatment [107]. This treatment has also shown curative effects in patients with ITP and human immunodeficiency virus infection [108, 109]. The ASGPR competitor asialofetuin may also improve platelet survival by attenuating platelet clearance in the liver [37].

Notwithstanding, there are still some unsolved questions that require further study. Previous reports have shown that Fc sialylation is a critical therapeutic target for immune system diseases such as inflammatory arthritis [110]. However, a recent report by Leonty D and colleagues showed that higher sialylated IVIG had no effect in their mouse model of ITP [111], which implied that the therapeutic effect is independent of sialylation. A recent study suggested that a novel neuraminidase inhibitor, peramivir, could not effectively increase the patients’ platelet count [112], which also argues against the curative effect of neuraminidase inhibition.

## Conclusions

As many studies have been conducted in recent years, our understanding of ITP has significantly improved. It has become increasingly clear that ITP is a highly complex autoimmune syndrome associated with autoantibodies, especially anti-GPIIb/IIIa and anti-GPIb complexes. Fc-FcγR interactions and the Fc-independent pathway are closely related to the progression of this condition, and recent studies have identified platelet desialylation as an essential part of the pathology of this disease. Desialylated platelets could be recognized by AMR and therefore destroyed in the liver. T cells, especially CD8 + T cells, are closely related to progression as well. Nevertheless, further research will be required to assess this novel concept. Recent studies have suggested the critical role of TFHs in the platelet desialylation of ITP. Treatments for ITP include traditional immunosuppressive and immunomodulatory therapies. In addition, splenectomy, RTX and TPO-receptor agonists can be effective. Desialylation may be a critical factor in refractory ITP, and sialylation inhibitors may be a potential treatment. ASGPR competitors may also be effective, yet further investigations of these unique therapeutic strategies are needed.

## Data Availability

Not applicable.

## Abbreviations

ITP: Immune thrombocytopenia
AMR: Ashwell-Morell receptor
GP: Glycoprotein
NEU1: Neuraminidase 1
TFHs: T follicular helper cells
PDW: Platelet distribution width
BM: Bone marrow
IVIG: Intravenous immunoglobulin G
MAbs: Monoclonal antibodies
LBD: Ligand binding domain
Tregs: Regulatory T cells
CTLs: Cytotoxic T lymphocytes
TPO: Thrombopoietin
MKs: Megakaryocytes
ASGPRs: Asialoglycoprotein receptors
VWF: von Willebrand factor
GT: Glanzmann thrombasthenia
PDGF: Platelet-derived growth factor
RTX: Rituximab
TPO-RAs: TPO-receptor agonists
IL-18 BP: IL-18 binding protein
DANA: 2-deoxy-2,3-didehydro-N-acetylneuraminic acid
MGL: Macrophage galactose lectin

## References

[1] Jones C, Denecke J, Strater R, Stolting T, Schunicht Y, Zeuschner D (2011). A novel type of macrothrombocytopenia associated with a defect in alpha2,3-sialylation. Am J Pathol.

[2] Gialluisi A, Izzi B, Di Castelnuovo A, Cerletti C, Donati MB, de Gaetano G (2020). Revisiting the link between platelets and depression through genetic epidemiology: new insights from platelet distribution width. Haematologica.

[3] Zufferey A, Kapur R, Semple JW (2017). Pathogenesis and Therapeutic Mechanisms in Immune Thrombocytopenia (ITP). J Clin Med.

[4] Cines DB, Bussel JB, Liebman HA, Luning Prak ET (2009). The ITP syndrome: pathogenic and clinical diversity. Blood.

[5] Zainal A, Salama A, Alweis R (2019). Immune thrombocytopenic purpura. J Community Hosp Intern Med Perspect.

[6] Kunishima S, Kamiya T, Saito H (2002). Genetic abnormalities of Bernard-Soulier syndrome. Int J Hematol.

[7] Seri M, Cusano R, Gangarossa S, Caridi G, Bordo D, Lo Nigro C (2000). Mutations in MYH9 result in the May-Hegglin anomaly, and Fechtner and Sebastian syndromes. The May-Heggllin/Fechtner Syndrome Consortium. Nat Genet.

[8] Willig TB, Breton-Gorius J, Elbim C, Mignotte V, Kaplan C, Mollicone R (2001). Macrothrombocytopenia with abnormal demarcation membranes in megakaryocytes and neutropenia with a complete lack of sialyl-Lewis-X antigen in leukocytes–a new syndrome?. Blood.

[9] Zhang L, Zhang M, Du X, Cheng Y, Cheng G (2020). Safety and efficacy of eltrombopag plus pulsed dexamethasone as first-line therapy for immune thrombocytopenia. Br J Haematol.

[10] Grozovsky R, Giannini S, Falet H, Hoffmeister KM (2015). Novel mechanisms of platelet clearance and thrombopoietin regulation. Curr Opin Hematol.

[11] Provan D, Stasi R, Newland AC, Blanchette VS, Bolton-Maggs P, Bussel JB (2010). International consensus report on the investigation and management of primary immune thrombocytopenia. Blood.

[12] Provan D, Newland A (2002). Fifty years of idiopathic thrombocytopenic purpura (ITP): management of refractory itp in adults. Br J Haematol.

[13] Zoghlami-Rintelen C, Weltermann A, Bittermann C, Kyrle PA, Pabinger I, Lechner K (2003). Efficacy and safety of splenectomy in adult chronic immune thrombocytopenia. Ann Hematol.

[14] Zeng Q, Zhu L, Tao L, Bao J, Yang M, Simpson EK (2012). Relative efficacy of steroid therapy in immune thrombocytopenia mediated by anti-platelet GPIIbIIIa versus GPIbalpha antibodies. Am J Hematol.

[15] McMillan R (2000). The pathogenesis of chronic immune (idiopathic) thrombocytopenic purpura. Semin Hematol.

[16] Stasi R, Evangelista ML, Stipa E, Buccisano F, Venditti A, Amadori S (2008). Idiopathic thrombocytopenic purpura: current concepts in pathophysiology and management. Thromb Haemost.

[17] Peng J, Ma SH, Liu J, Hou Y, Liu XM, Niu T (2014). Association of autoantibody specificity and response to intravenous immunoglobulin G therapy in immune thrombocytopenia: a multicenter cohort study. J Thromb Haemost.

[18] Quach ME, Chen W, Li R (2018). Mechanisms of platelet clearance and translation to improve platelet storage. Blood.

[19] Li J, Sullivan, He N (2018). Pathophysiology of immune thrombocytopenia. Curr Opin Hematol.

[20] Okumura I, Lombart C, Jamieson GA (1976). Platelet glycocalicin. II. Purification and characterization. J Biol Chem.

[21] Urbanus RT, van der Wal DE, Koekman CA, Huisman A, van den Heuvel DJ, Gerritsen HC (2013). Patient autoantibodies induce platelet destruction signals via raft-associated glycoprotein Ibalpha and Fc RIIa in immune thrombocytopenia. Haematologica.

[22] Li C, Piran S, Chen P, Lang S, Zarpellon A, Jin J (2011). The maternal immune response to fetal platelet GPIbα causes frequent miscarriage in mice that can be prevented by intravenous IgG and anti-FcRn therapies. J Clin Invest.

[23] Zhao X, Qi X, Wang C, Zhou Z, Cao H, Wu P (2017). Idiopathic thrombocytopenic purpura: pathogenesis and potential therapeutic approach. Minerva Med.

[24] Hu Y, Li H, Zhang L, Shan B, Xu X, Li H (2012). Elevated profiles of Th22 cells and correlations with Th17 cells in patients with immune thrombocytopenia. Hum Immunol.

[25] Nishimoto T, Kuwana M (2013). CD4 + CD25 + Foxp3 + Regulatory T Cells in the Pathophysiology of Immune Thrombocytopenia. Semin Hematol.

[26] Yu J, Heck S, Patel V, Levan J, Yu Y, Bussel JB (2008). Defective circulating CD25 regulatory T cells in patients with chronic immune thrombocytopenic purpura. Blood.

[27] Panitsas FP, Theodoropoulou M, Kouraklis A, Karakantza M, Theodorou GL, Zoumbos NC (2004). Adult chronic idiopathic thrombocytopenic purpura (ITP) is the manifestation of a type-1 polarized immune response. Blood.

[28] McKenzie CG, Guo L, Freedman J, Semple JW (2013). Cellular immune dysfunction in immune thrombocytopenia (ITP). Br J Haematol.

[29] Olsson B, Ridell B, Carlsson L, Jacobsson S, Wadenvik H (2008). Recruitment of T cells into bone marrow of ITP patients possibly due to elevated expression of VLA-4 and CX3CR1. Blood.

[30] Semple JW, Italiano JE, Freedman J (2011). Platelets and the immune continuum. Nat Rev Immunol.

[31] Cooper N, Bussel J (2006). The pathogenesis of immune thrombocytopaenic purpura. Br J Haematol.

[32] Consolini R, Legitimo A, Caparello MC (2016). The centenary of immune thrombocytopenia - part 1: revising nomenclature and pathogenesis. Front Pediatr.

[33] Olsson B, Andersson P-O, Jernås M, Jacobsson S, Carlsson B, Carlsson LMS (2003). T-cell-mediated cytotoxicity toward platelets in chronic idiopathic thrombocytopenic purpura. Nat Med.

[34] Iraqi M, Perdomo J, Yan F, Choi PY, Chong BH (2015). Immune thrombocytopenia: antiplatelet autoantibodies inhibit proplatelet formation by megakaryocytes and impair platelet production in vitro. Haematologica.

[35] Zufferey A, Speck ER, Machlus KR, Aslam R, Guo L, McVey MJ (2017). Mature murine megakaryocytes present antigen-MHC class I molecules to T cells and transfer them to platelets. Blood Adv.

[36] Li S, Wang L, Zhao C, Li L, Peng J, Hou M (2007). CD8 + T cells suppress autologous megakaryocyte apoptosis in idiopathic thrombocytopenic purpura. Br J Haematol.

[37] Qiu J, Liu X, Li X, Zhang X, Han P, Zhou H (2016). CD8 + T cells induce platelet clearance in the liver via platelet desialylation in immune thrombocytopenia. Sci Rep.

[38] Crotty S (2014). T follicular helper cell differentiation, function, and roles in disease. Immunity.

[39] Baumjohann D, Preite S, Reboldi A, Ronchi F, Ansel KM, Lanzavecchia A (2013). Persistent antigen and germinal center B cells sustain T follicular helper cell responses and phenotype. Immunity.

[40] Yusuf I, Stern J, McCaughtry TM, Gallagher S, Sun H, Gao C (2014). Germinal center B cell depletion diminishes CD4 + follicular T helper cells in autoimmune mice. PloS one.

[41] Audia S, Rossato M, Trad M, Samson M, Santegoets K, Gautheron A (2017). B cell depleting therapy regulates splenic and circulating T follicular helper cells in immune thrombocytopenia. J Autoimmun.

[42] Yao X, Li C, Yang J, Wang G, Li C, Xia Y (2016). Differences in frequency and regulation of T follicular helper cells between newly diagnosed and chronic pediatric immune thrombocytopenia. Blood Cells Mol Dis.

[43] Xie J, Cui D, Liu Y, Jin J, Tong H, Wang L (2015). Changes in follicular helper T cells in idiopathic thrombocytopenic purpura patients. Int J Biol Sci.

[44] Kile BT (2014). The role of apoptosis in megakaryocytes and platelets. Br J Haematol.

[45] Justo Sanz R, Monzon Manzano E, Fernandez Bello I, Teresa Alvarez Roman M, Martin Salces M, Rivas Pollmar MI (2019). Platelet Apoptosis and PAI-1 are Involved in the Pro-Coagulant State of Immune Thrombocytopaenia Patients Treated with Thrombopoietin Receptor Agonists. Thromb Haemost.

[46] Grodzielski M, Goette NP, Glembotsky AC, Constanza Baroni Pietto M, Mendez-Huergo SP, Pierdominici MS (2019). Multiple concomitant mechanisms contribute to low platelet count in patients with immune thrombocytopenia. Sci Rep.

[47] Engel C, Loeffler M, Franke H, Schmitz S (1999). Endogenous thrombopoietin serum levels during multicycle chemotherapy. Br J Haematol.

[48] Xu M, Li J, Neves MAD, Zhu G, Carrim N, Yu R (2018). GPIbα is required for platelet-mediated hepatic thrombopoietin generation. Blood.

[49] Ng AP, Kauppi M, Metcalf D, Hyland CD, Josefsson EC, Lebois M (2014). Mpl expression on megakaryocytes and platelets is dispensable for thrombopoiesis but essential to prevent myeloproliferation. Proc Natl Acad Sci U S A.

[50] Meyer SC, Keller MD, Woods BA, LaFave LM, Bastian L, Kleppe M (2014). Genetic studies reveal an unexpected negative regulatory role for Jak2 in thrombopoiesis. Blood.

[51] Hoffmeister KM, Falet H (2016). Platelet clearance by the hepatic Ashwell-Morrell receptor: mechanisms and biological significance. Thromb Res.

[52] Li MF, Li XL, Fan KL, Yu YY, Gong J, Geng SY (2017). Platelet desialylation is a novel mechanism and a therapeutic target in thrombocytopenia during sepsis: an open-label, multicenter, randomized controlled trial. J Hematol Oncol.

[53] Goswami K, Koner BC (2002). Level of sialic acid residues in platelet proteins in diabetes, aging, and Hodgkin’s lymphoma: a potential role of free radicals in desialylation. Biochem Biophys Res Commun.

[54] Grewal PK, Aziz PV, Uchiyama S, Rubio GR, Lardone RD, Le D (2013). Inducing host protection in pneumococcal sepsis by preactivation of the Ashwell-Morell receptor. Proc Natl Acad Sci U S A.

[55] Zhang X-H, Wang Q-M, Zhang J-M, Feng F-E, Wang F-R, Chen H (2015). Desialylation is associated with apoptosis and phagocytosis of platelets in patients with prolonged isolated thrombocytopenia after allo-HSCT. J Hematol Oncol.

[56] van der Wal DE, Du VX, Lo KS, Rasmussen JT, Verhoef S, Akkerman JW (2010). Platelet apoptosis by cold-induced glycoprotein Ibalpha clustering. J Thromb Haemost.

[57] Cho J, Kim H, Song J, Cheong JW, Shin JW, Yang WI (2018). Platelet storage induces accelerated desialylation of platelets and increases hepatic thrombopoietin production. J Transl Med.

[58] Revilla N, de la Morena-Barrio ME, Miñano A, López-Gálvez R, Toderici M, Padilla J (2017). Transient desialylation in combination with a novel antithrombin deficiency causing a severe and recurrent thrombosis despite anticoagulation therapy. Sci Rep.

[59] Stivala S, Gobbato S, Infanti L, Reiner MF, Bonetti N, Meyer SC (2017). Amotosalen/ultraviolet A pathogen inactivation technology reduces platelet activatability, induces apoptosis and accelerates clearance. Haematologica.

[60] Ellies LG, Sperandio M, Underhill GH, Yousif J, Smith M, Priatel JJ (2002). Sialyltransferase specificity in selectin ligand formation. Blood.

[61] Revilla N, Corral J, Minano A, Mingot-Castellano ME, Campos RM, Velasco F (2019). Multirefractory primary immune thrombocytopenia; targeting the decreased sialic acid content. Platelets.

[62] Li R, Hoffmeister KM, Falet H (2016). Glycans and the platelet life cycle. Platelets.

[63] Dupont A, Soukaseum C, Cheptou M, Adam F, Nipoti T, Lourenco-Rodrigues MD (2019). Relevance of platelet desialylation and thrombocytopenia in type 2B von Willebrand disease: preclinical and clinical evidence. Haematologica.

[64] Li J, Callum JL, Lin Y, Zhou Y, Zhu G, Ni H (2014). Severe platelet desialylation in a patient with glycoprotein Ib/IX antibody-mediated immune thrombocytopenia and fatal pulmonary hemorrhage. Haematologica.

[65] Li J, van Der Wal DE, Zhu G, Xu M, Issaka Y, Ma L (2014). Platelet desialylation: A Novel mechanism of Fc-independent platelet clearance and a potential diagnostic biomarker and therapeutic target in immune thrombocytopenia. Blood.

[66] Grewal PK, Uchiyama S, Ditto D, Varki N, Le DT, Nizet V (2008). The Ashwell receptor mitigates the lethal coagulopathy of sepsis. Nat Med.

[67] Grewal PK (2010). The Ashwell-Morell receptor. Methods Enzymol.

[68] Li Y, Fu J, Ling Y, Yago T, McDaniel JM, Song J (2017). Sialylation on O-glycans protects platelets from clearance by liver Kupffer cells. Proc Natl Acad Sci U S A.

[69] Deppermann C, Kratofil RM, Peiseler M, David BA, Zindel J, Castanheira FVES (2020). Macrophage galactose lectin is critical for Kupffer cells to clear aged platelets. J Exp Med.

[70] Jansen AJG, Josefsson EC, Rumjantseva V, Liu QP, Falet H, Bergmeier W (2012). Desialylation accelerates platelet clearance after refrigeration and initiates GPIbα metalloproteinase-mediated cleavage in mice. Blood.

[71] Deng W, Xu Y, Chen W, Paul DS, Syed AK, Dragovich MA (2016). Platelet clearance via shear-induced unfolding of a membrane mechanoreceptor. Nat Commun.

[72] Riswari SF, Tunjungputri RN, Kullaya V, Garishah FM, Utari GSR, Farhanah N (2019). Desialylation of platelets induced by Von Willebrand Factor is a novel mechanism of platelet clearance in dengue. PLoS Pathog.

[73] Grozovsky R, Giannini S, Falet H, Hoffmeister KM (2015). Regulating billions of blood platelets: glycans and beyond. Blood.

[74] Kullaya V, de Jonge MI, Langereis JD, van der Gaast-de Jongh CE, Bull C, Adema GJ (2018). Desialylation of platelets by pneumococcal neuraminidase A Induces ADP-dependent platelet hyperreactivity. Infect Immun..

[75] van der Wal DE, Davis AM, Mach M, Marks DC (2020). The role of neuraminidase 1 and 2 in glycoprotein Ibalpha-mediated integrin alphaIIbbeta3 activation. Haematologica.

[76] Zheng SS, Perdomo JS, Leung HHL, Yan F, Chong BH (2020). Acquired Glanzmann thrombasthenia associated with platelet desialylation. J Thromb Haemost.

[77] Perdicchio M, Ilarregui JM, Verstege MI, Cornelissen LA, Schetters ST, Engels S (2016). Sialic acid-modified antigens impose tolerance via inhibition of T-cell proliferation and de novo induction of regulatory T cells. Proc Natl Acad Sci U S A.

[78] Monzon Manzano E, Alvarez Roman MT, Justo Sanz R, Fernandez Bello I, Hernandez D, Martin Salces M (2020). Platelet and immune characteristics of immune thrombocytopaenia patients non-responsive to therapy reveal severe immune dysregulation. Br J Haematol.

[79] Ma X, Li Y, Kondo Y, Shi H, Han J, Jiang Y (2020). Slc35a1 deficiency causes thrombocytopenia due to impaired megakaryocytopoiesis and excessive platelet clearance in the liver. Haematologica.

[80] Grozovsky R, Begonja AJ, Liu K, Visner G, Hartwig JH, Falet H (2015). The Ashwell-Morell receptor regulates hepatic thrombopoietin production via JAK2-STAT3 signaling. Nat Med.

[81] Tinazzi E, Osti N, Beri R, Argentino G, Veneri D, Dima F (2020). Pathogenesis of immune thrombocytopenia in common variable immunodeficiency. Autoimmun Rev.

[82] LeVine DN, Brooks MB (2019). Immune thrombocytopenia (ITP): Pathophysiology update and diagnostic dilemmas. Vet Clin Pathol.

[83] Hinek A, Bodnaruk TD, Bunda S, Wang Y, Liu K (2008). Neuraminidase-1, a subunit of the cell surface elastin receptor, desialylates and functionally inactivates adjacent receptors interacting with the mitogenic growth factors PDGF-BB and IGF-2. Am J Pathol.

[84] Marini I, Zlamal J, Faul C, Holzer U, Hammer S, Pelzl L (2019). Autoantibody-mediated desialylation impairs human thrombopoiesis and platelet life span. Haematologica.

[85] Cantoni S, Carpenedo M, Nichelatti M, Sica L, Rossini S, Milella M (2016). Clinical relevance of antiplatelet antibodies and the hepatic clearance of platelets in patients with immune thrombocytopenia. Blood.

[86] Samson M, Fraser W, Lebowitz D (2019). Treatments for primary immune thrombocytopenia: a review. Cureus.

[87] Cheng Y, Wong RS, Soo YO, Chui CH, Lau FY, Chan NP (2003). Initial treatment of immune thrombocytopenic purpura with high-dose dexamethasone. N Engl J Med.

[88] Nomura S (2016). Advances in diagnosis and treatments for immune thrombocytopenia. Clin Med Insights Blood Disord.

[89] Mayer B, Depré F, Ringel F, Salama A (2017). New aspects on the efficacy of high-dose intravenous immunoglobulins in patients with autoimmune thrombocytopenia. Vox Sang.

[90] Al Askar AS, Shaheen NA, Al Zahrani M, Al Otaibi MG, Al Qahtani BS, Ahmed F (2018). Splenectomy vs. rituximab as a second-line therapy in immune thrombocytopenic purpura: a single center experience. Int J Hematol.

[91] Kuwana M, Okazaki Y, Kaburaki J, Kawakami Y, Ikeda Y (2002). Spleen is a primary site for activation of platelet-reactive T and B cells in patients with immune thrombocytopenic purpura. J Immunol.

[92] Newland A, Godeau B, Priego V, Viallard JF, Lopez Fernandez MF, Orejudos A (2016). Remission and platelet responses with romiplostim in primary immune thrombocytopenia: final results from a phase 2 study. Br J Haematol.

[93] Vianelli N, Galli M, de Vivo A, Intermesoli T, Giannini B, Mazzucconi MG (2005). Efficacy and safety of splenectomy in immune thrombocytopenic purpura: long-term results of 402 cases. Haematologica.

[94] Fujimura K, Miyakawa Y, Kurata Y, Kuwana M, Tomiyama Y, Murata M (2012). [Reference guide for management of adult idiopathic thrombocytopenic purpura (ITP) 2012 version]. Rinsho Ketsueki.

[95] Lambert MP, Gernsheimer TB (2017). Clinical updates in adult immune thrombocytopenia. Blood.

[96] Cooper N (2017). State of the art - how I manage immune thrombocytopenia. Br J Haematol.

[97] H. Mei, X. Liu, Y. Li, H. Zhou, Y. Feng, G. Gao, et al. (2021) A multicenter, randomized phase III trial of hetrombopag: a novel thrombopoietin receptor agonist for the treatment of immune thrombocytopenia. J Hematol Oncol. 14(1). 10.1186/s13045-021-01047-9 PMC7905908 33632264

[98] Yazdanbakhsh K (2016). TPO-RAs multitask in ITP. Blood.

[99] Kapur R, Aslam R, Speck ER, Rebetz JM, Semple JW (2020). Thrombopoietin receptor agonist (TPO-RA) treatment raises platelet counts and reduces anti-platelet antibody levels in mice with immune thrombocytopenia (ITP). Platelets.

[100] Bao W, Bussel JB, Heck S, He W, Karpoff M, Boulad N (2010). Improved regulatory T-cell activity in patients with chronic immune thrombocytopenia treated with thrombopoietic agents. Blood.

[101] Neunert C, Lim W, Crowther M, Cohen A, Solberg L, Crowther MA (2011). The American Society of Hematology 2011 evidence-based practice guideline for immune thrombocytopenia. Blood.

[102] Wei Y, Hou M (2016). T cells in the pathogenesis of immune thrombocytopenia. Semin Hematol.

[103] Li J, Tian J, Lu J, Wang Z, Ling J, Wu X (2020). LncRNA GAS5 inhibits Th17 differentiation and alleviates immune thrombocytopenia via promoting the ubiquitination of STAT3. Int Immunopharmacol.

[104] Kaufman RM, Djulbegovic B, Gernsheimer T, Kleinman S, Tinmouth AT, Capocelli KE (2015). Platelet transfusion: a clinical practice guideline from the AABB. Ann Intern Med.

[105] Li J, van der Wal DE, Zhu G, Xu M, Yougbare I, Ma L (2015). Desialylation is a mechanism of Fc-independent platelet clearance and a therapeutic target in immune thrombocytopenia. Nat Commun.

[106] Shao L, Wu Y, Zhou H, Qin P, Ni H, Peng J (2015). Successful treatment with oseltamivir phosphate in a patient with chronic immune thrombocytopenia positive for anti-GPIb/IX autoantibody. Platelets.

[107] Shaim H, McCaffrey P, Trieu JA, DeAnda A, Yates SG (2020). Evaluating the effects of oseltamivir phosphate on platelet counts: a retrospective review. Platelets..

[108] Alvarez-Roman MT, Rivas Pollmar MI, Bernardino JI, Lozano ML, Martin-Salces M, Fernandez-Bello I (2016). Thrombopoietin receptor agonists in conjunction with oseltamivir for immune thrombocytopenia. AIDS.

[109] Bigot P, Auffret M, Gautier S, Weinborn M, Ettahar NK, Coupe P (2016). Unexpected platelets elevation in a patient with idiopathic thrombocytopenia treated with oseltamivir for influenza infection. Fundam Clin Pharmacol.

[110] Anthony RM, Ravetch JV (2010). A novel role for the IgG Fc glycan: the anti-inflammatory activity of sialylated IgG Fcs. J Clin Immunol.

[111] Leontyev D, Katsman Y, Ma XZ, Miescher S, Kasermann F, Branch DR (2012). Sialylation-independent mechanism involved in the amelioration of murine immune thrombocytopenia using intravenous gammaglobulin. Transfusion.

[112] Kim YG, Ko SY, Lee SW (2019). Comparison of the effects of peramivir and oseltamivir on the rise in platelet count in patients with or without proven influenza. Int J Clin Pharmacol Ther.

